# Dr. Harry Heath Rodman

**Published:** 1906-12

**Authors:** 


Dr. Harry Heath Rodman, of Buffalo, formerly of New York, died by his own hand while in a temporary fit of despondency, November 12, 1906. He was a native of Brooklyn and graduated from the College of Physicians and Surgeons, Medical Department of Columbia University, in 1895. He recently had an attack of scarlet fever, which left his health impaired and affected his hearing. He submitted to several operations without benefit, and the prospects of ultimate deafness is believed to have so preyed upon his mind as to have prompted him to self destruction. He was a man of promise and leaves a wife in great bereavement.

